# Unexpected Death of a Sickle Cell Disease Patient: A Case Report and Literature Review

**DOI:** 10.7759/cureus.62645

**Published:** 2024-06-18

**Authors:** Gang Zhou, Amin Mohammad, Yuan Shan

**Affiliations:** 1 Pathology, Baylor Scott & White Medical Center-Temple, Temple, USA

**Keywords:** clinical pathology, vaso-occlusive crisis, apheresis, nephropathy, sickle cell disease

## Abstract

Sickle cell disease (SCD) is an autosomal recessive genetic disorder characterized by the abnormal formation of sickle hemoglobin (HbS). Under conditions of deoxygenation, HbS undergoes polymerization, resulting in microvascular occlusion, tissue hypoxia, and infarction. The elevated mortality rate associated with SCD is primarily attributed to complications such as sepsis, acute chest syndrome, stroke, acute multiorgan failure, and pulmonary hypertension. Despite advancements in awareness and treatments, preventing mortality in young individuals with SCD remains a formidable challenge. In an effort to shed light on these challenges, we present a case of unexpected death associated with SCD to emphasize the pressing need for continued research and intervention strategies to improve patient outcomes.

## Introduction

Sickle cell disease (SCD), an inherited hemoglobin disorder, is characterized by mutated βS-globin chains forming hemoglobin S (HbS), causing red blood cells (RBCs) to deform into sickle shapes when deoxygenated. This leads to membrane damage, altered blood rheology, and intravascular hemolysis, triggering an inflammatory cascade affecting Virchow’s triad. It affects around 300,000 infants annually [1], with a notable presence in various regions, including sub-Saharan Africa, India, the Mediterranean, Middle East, and 100,000 cases in the United States (US). Diagnosis involves newborn screening or identification during episodes of severe pain or normocytic anemia. SCD is primarily associated with mortality resulting from infection, pain episodes, acute chest syndrome (ACS), and stroke [2,3]. In sickle cell anemia, death can occur suddenly and unexpectedly [2]. A prevalent and life-threatening manifestation is the vaso-occlusive crisis (VOCs), often leading to fatal outcomes [4]. VOCs result from RBC adhesion to postcapillary venule walls. Susceptibility to acute VOCs is heightened by chronic inflammation, cardiopulmonary compromise, and renal dysfunction. Managing acute crises is challenging due to the cardiovascular system's inability to cope with sudden oxygen demand increases and concurrent renal dysfunction [5]. Although most children with SCD reach adulthood in the US, their life expectancy is typically 20 years shorter than the general population [6].

## Case presentation

A 36-year-old male known to the Hematology/Oncology Clinic for hemoglobin (Hgb) SS SCD, presented to the emergency department (ED) on day 1 with a three-day history of severe sickle cell pain crisis in both upper and lower extremities. Home remedies and morphine every three hours provided no relief. Following a crizanlizumab infusion, symptoms acutely worsened, including substernal chest pain and dyspnea. ED evaluation showed constant, sharp chest pain exacerbated by palpation or deep inspiration. No associated symptoms like nausea, vomiting, fever, chills, or cough were reported. Abdominal discomfort and decreased urine output were noted. Improvement was reported after Dilaudid 4 mg intravenous (IV), but the patient remains uncomfortable. Initial vital signs included a temperature of 97.7 Fahrenheit (F), heart rate of 116/min (minute), respiratory rate of 22/min, oxygen saturation of 93%, and blood pressure of 171/102 millimeters of mercury (mm Hg). Laboratory findings indicated abnormalities in white blood cells (WBC), Hgb, hematocrit, carbon dioxide (CO2), glucose, total bilirubin, alkaline phosphatase, aspartate aminotransferase (AST), and reticulocytes (Table 1).

**Table 1:** Laboratory results WBC: White blood cell; RBC: red blood cell; BUN: blood urea nitrogen; MCV: mean corpuscular volume; MCH: mean corpuscular hemoglobin; MCHC: mean corpuscular hemoglobin concentration; RDW: red cell distribution width; AST: aspartate aminotransferase; ALT: alanine aminotransferase; NRBC: nucleated red blood cell; HDL: high-density lipoprotein; LDL: low-density lipoprotein; eGFR: estimated glomerular filtration rate; BASO: basophils; EOS: eosinophils; GRAN: granulocytes

Chemistry				Hematology			
Tests	Day 1	Day 2	Reference range & units	Tests	Day 1	Day 2	Reference range & units
Glucose	107	69	70-100 mg/dL	WBC	19.3	9.2	4.8-10.8 X 10^9^/L
BUN	11	15	7-22 mg/dL	RBC	3.17	1.58	4.70-6.10 X 10^12^/L
Creatinine	0.63	0.67	0.50-1.30 mg/dL	Hemoglobin	7.7	3.8	14.0-18.0 g/dL
Sodium	140	148	136-145 meq/L	Hematocrit	22.0	11.5	42.0-52.0 %
Potassium	4.5	4.1	3.5-5.3 meq/L	MCV	69.4	72.8	80.0-94.0 fL
Chloride	111	121	97-111 meq/L	MCH	24.3	24.1	27.0-34.5 pg
Carbon dioxide	20	7	22-30 meq/L	MCHC	35.0	33.0	32.0-36.5 g/dL
Calcium	8.5	5.2	8.6-10.5 mg/dL	RDW	24.2	25.4	11.0-15.0%
Total bilirubin	9.6	6.6	0.2-1.2 mg/dL	Platelet	118	57	150-450 10^9^/L
Alkaline phosphatase	191	210	34-130 IU/L	GRAN%	87	47	%
AST	166	195	0-40 IU/L	BAND%	1	-	%
ALT	18	42	0-68 IU/L	LYMPH%	9	43	%
Total protein	6.1	3.3	6.0-8.0 g/dL	MONO%	2	3	%
Albumin	3.1	1.7	3.5-5.2 g/dL	EOS%	0	0	%
Anion gap	11	20	4-16 meq/L	BASO%	0	1	%
Phosphorus	4.1	6.4	2.4-4.2 mg/dL	Metamyelocytes%	1	5	%
Magnesium	2.0	3.3	1.8-2.4 mg/dL	Myelocytes%	-	1	%
Cholesterol	-	62	mg/dL	NRBC	91.0	117.0	0.0-0.9/100 WBC
Triglycerides	-	89	mg/dL				
HDL	-	12	40-60 mg/dL				
LDL	-	32	mg/dL				
Ionized calcium	4.90	-	4.70-5.20 mg/dL				
Troponin I	1.70	1.92	0.00-0.09 ng/mL				
eGFR	126	124	≥60 mL/min/1.73 m^2^				

**Figure 1:**
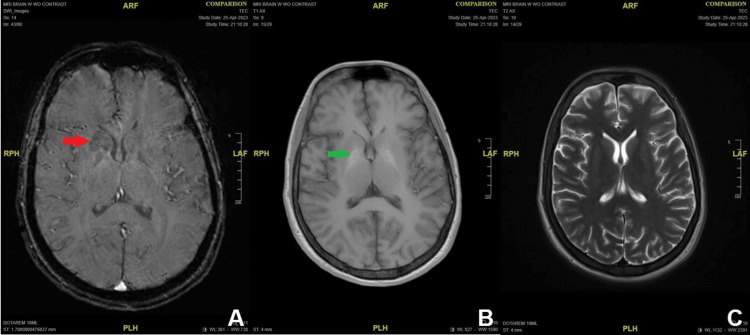
Magnetic resonance imaging (A) Mild restricted diffusion (red arrow) in the right caudate head extending into the anterior limb of internal capsule and the adjacent lentiform nucleus in susceptibility-weighted imaging (SWI). (B) Mild intrinsic globus pallidus hyperintensity (green arrow) in T1-weighted imaging. (C) Subtle associated asymmetric hyperintensity T2-weighted imaging

Electrocardiogram (EKG) showed sinus tachycardia and nonspecific ST changes. The patient received multiple pain medications and IV fluids in the ED and was admitted to the Heme/Onc team for further evaluation and work-up for systemic inflammatory response syndrome (SIRS). An RBC exchange was scheduled. Regrettably, the patient's condition deteriorated rapidly, with significant drops in RBC, Hgb, albumin, and platelets (Table 1). Upon presentation to the intensive care unit (ICU) on day 2, the patient experienced progressive respiratory distress, worsening hypertension, and elevated heart rate. Susceptibility-weighted imaging (SWI) demonstrates mild restricted diffusion in the right caudate head extending into the anterior limb of internal capsule and the adjacent lentiform nucleus (Figure 1A). T1-weighted imaging shows mild intrinsic globus pallidus hyperintensity (Figure 1B). T2-weighted imaging shows subtle associated asymmetric hyperintensity (Figure 1C).

**Figure 2:**
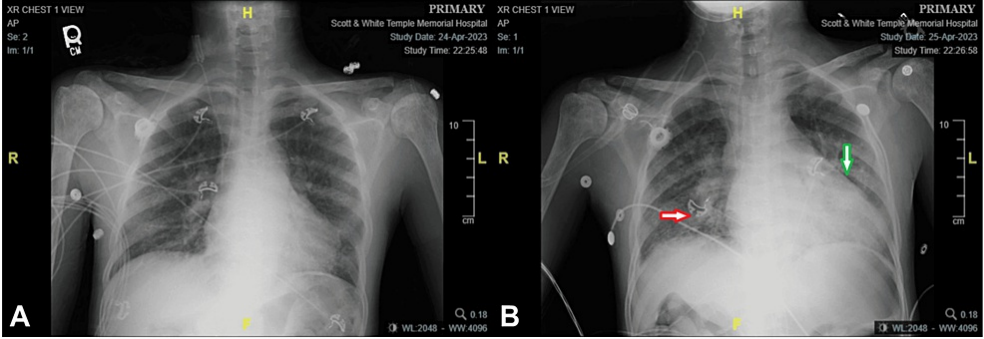
Chest X-ray demonstrates the development of interstitial and alveolar opacities in both mid to lower lung zones (red arrow) and enlarged cardiac silhouette (green arrow) in day 2 (B) in comparison to day 1 (A)

Chest X-ray demonstrates the development of interstitial and alveolar opacities in both mid to lower lung zones and enlarged cardiac silhouette (Figure 2B) in comparison to day 1 (Figure 2A).

Despite immediate intervention, including chest compressions and administration of tissue plasminogen activator (tPA), pulse restoration was unsuccessful. In consultation with the family, the medical team decided to withdraw care. The patient passed away soon after.

## Discussion

Individuals with SCD face a heightened risk of multiple severe comorbid conditions. The life span is believed to be 20 years shorter than the general population and decreased by 25 to 30 years in the African American population [7]. The ACS is the leading cause of death in patients >10 years of age. ACS, one of the manifestations of VOCs, involves a sudden drop in oxygen saturation and is marked by a new chest X-ray infiltrate. Common symptoms include fever, tachypnea, coughing, and chest pain. It is often linked to RBC sickling and can be idiopathic or associated with factors like infection, pulmonary infarction, or fat embolism.

The patient presented in the ED with acute pain syndrome. Although neuropathic pain can also be observed, acute pain is mostly related to vaso-occlusion. Inadequate pain management can lead to complications like respiratory splinting, potentially causing hypoxemia and increased sickling, thereby increasing the risk of conditions such as ACS or other complications related to SCD, such as stroke and nephropathy. The patient experienced a rapid decline in RBC (from 3.17 to 1.58 X 10^12/L), albumin (from 3.1 to 1.7 g/L), and total protein (from 6.1 to 3.3 g/L) within 24 hours, coupled with an elevated urine protein level exceeding 300 mg/dL. These findings suggest acute kidney dysfunction possibly caused by mixed glomerular and tubular injuries. Hypoproteinemia in the patient could be linked to pulmonary edema and pericardial effusion (Figure 2B), intensifying his ACS. Manifesting tachycardia, tachypnea, and respiratory acidosis (Table 1), he experienced heightened hypoxia and RBC sickling in the pulmonary vascular space. Furthermore, his underlying SIRS compromised vascular integrity, coupled with low colloid osmotic pressure, exacerbating pulmonary edema and raising the risk of acute respiratory distress syndrome (ARDS). Neurological dysfunction frequently arises in SCD, manifesting as acute cerebrovascular accidents, headaches, seizures, or cognitive decline [8]. The patient’s worsening conditions prompted an MRI, revealing asymmetric hyperintensity and restricted diffusion in the right basal ganglia (BG) (Figure 1), signifying acute ischemic changes. Reported findings indicate reduced tracer uptake in the BG among SCD patients [9], attributed to the vulnerability of specialized capillaries to sickled RBCs and intimal hyperplasia in large cerebral arteries [10], ultimately contributing to BG infarction. In addition, his low HDL level (12 mg/dL) and high troponin I level (1.92 ng/mL) may indicate a possible underlying cardiovascular and ischemic heart disease. However, the troponin level is difficult to interpret in the setting of acute renal failure and acute stroke. Pulmonary arterial hypertension (PAH) is a significant complication and independent risk factor for sudden death in adults with SCD, suggesting a potential link between apolipoproteins and PAH vasculopathy, possibly paralleling atherosclerosis mechanistically [11]. Severe ACS is associated with elevated pulmonary pressures which is linked to increased levels of cardiac biomarkers, such as troponin I [12], and an increased risk of mortality [13]. These consequences could be averted through proactive RBC exchange. While current category I and II indications are acute stroke and severe ACS [14], waiting until VOCs have begun would be too late to reverse the patients' catastrophic decline. Thus, prompt prophylaxis RBC exchange is more important. RBC exchange shows promise in improving blood viscosity and vessel relaxation time and reducing adhesion molecules like vascular cell adhesion molecule 1 (VCAM-1). Guilliams [15] suggests it may also lower cerebral blood flow, decrease oxygen extraction, and mitigate cerebral metabolic stress, potentially reducing the risk of infarction.

Sickle cell anemia and related hemoglobinopathies are associated with various renal abnormalities. Individuals who quickly advance to nephrotic-range proteinuria exhibit a decline in kidney function. These include compromised urinary concentrating ability, difficulties in urinary acidification and potassium excretion, and supernormal proximal tubular function, as indicated by elevated creatinine secretion and glomerular filtration rate (GFR). Thus, serum cystatin C is suggested to be more sensitive than serum creatinine for detecting early declines in kidney function [16]. As noticed in this patient, his eGFR was 124 mL/min/1.73 m2 even with significant proteinuria (Table 1). Among adults with SCD, proteinuria, potentially advancing to nephrotic syndrome, serves as a predictor for the risk of end-stage renal disease (ESRD), alongside hypertension and escalating anemia. Prevalence rates indicate proteinuria in 20-25% of SCD patients and 8-30% of individuals with other hemoglobinopathies [17].

Diagnosis of renal disease secondary to SCD relies mainly on clinical manifestations and is primarily a diagnosis of exclusion [18]. Close monitoring is advised for patients with albuminuria to detect progression, and initiating therapy with hydroxyurea and/or angiotensin blockade is recommended for those developing proteinuria. Initiate angiotensin-converting enzyme inhibitor (ACEI) or angiotensin II receptor blocker (ARB) therapy in proteinuria patients, irrespective of baseline hypertension, as it can reduce protein excretion by up to 50% [19]. In order to slow the progression of sickle cell nephropathy to ESRD, it is crucial to effectively manage hypertension and proteinuria. However, to truly prevent the renal complications associated with SCD, a cure for this genetic disorder will be essential [20].

## Conclusions

Sickle cell crisis affects multiple organ systems and can lead to permanent impairment once initiated. A strategic approach to managing SCD involves preventing VOCs. RBC exchange stands out as a key prophylactic and therapeutic measure to decrease SCD mortality. Monitoring sickle cell nephropathy can serve as an indicator, offering insights into when to initiate RBC exchange.
